# Latent autoimmune diabetes in an adult patient presenting as suspected neuroendocrine tumour, Crohn's disease and malabsorption syndrome: A case report

**DOI:** 10.3892/mi.2026.342

**Published:** 2026-09-29

**Authors:** Ami Bhalodia, Prachi Patel, Rand Daoud, Parth Adrejiya, Nishi Patel, Khemaporn Lertdetkajorn

**Affiliations:** 1 Department of Internal Medicine, Hamilton Medical Center, Dalton, GA 30720, USA; 2 Department of Internal Medicine, Southern Regional Medical Center, Riverdale, GA 30274, USA; 3 Department of Internal Medicine, Wellstar Cobb Medical Center, Austell, GA 30106, USA; 4 Department of Internal Medicine, Wellstar Spalding Medical Center, Griffin, GA 30224, USA; 5 Department of Internal Medicine, Athabasca University, Athabasca, Alberta T9S 3A3, Canada; 6 Department of Endocrinology, Southern Regional Medical Center, Riverdale, GA 30274, USA

**Keywords:** latent autoimmune diabetes in adults, autoimmune diabetes, hyperosmolar hyperglycaemic state, islet autoantibodies, C-peptide deficiency, insulin therapy

## Abstract

Latent autoimmune diabetes in adults (LADA) is an autoimmune form of diabetes frequently misclassified as type 2 diabetes mellitus (T2DM), resulting in delayed insulin initiation and the progressive loss of β-cells. The present case report describes the case of a 39-year-old man with long-standing, severely uncontrolled diabetes who presented with hyperosmolar hyperglycaemic state (HHS), profound unintentional weight loss (113.4 to 57.2 kg over a period of 1 year), chronic watery diarrhoea and a pruritic vesicular rash. This constellation prompted an evaluation for neuroendocrine tumour, Crohn's disease, and malabsorption syndromes before the underlying autoimmune diabetes was recognised. Extensive infectious, malignant and gastrointestinal evaluations were unrevealing. Endocrine workup demonstrated positive glutamic acid decarboxylase (GAD-65, 33 IU/ml; reference, <5), islet cell (1:8; reference, <1:4) and zinc transporter 8 (ZnT8, 20 U/ml; reference, <10) antibodies with severely reduced C-peptide (0.12 nmol/l; reference, 0.26-1.28), confirming LADA. He was managed with intravenous insulin for HHS and transitioned to neutral protamine Hagedorn (NPH) and regular insulin, with improved glycaemic control and partial symptom resolution. The present case report highlights the diagnostic challenges of LADA in adults presumed to have T2DM despite poor glycaemic control, and demonstrates that profound insulin deficiency can phenocopy malignancy and inflammatory bowel disease. Recognition through islet autoantibody and C-peptide testing is essential for timely insulin initiation, which may limit hyperglycaemia, β-cell decline and complications.

## Introduction

Latent autoimmune diabetes in adults (LADA) is an adult-onset form of autoimmune diabetes combining features of both type 1 and type 2 diabetes. Defined by islet autoantibodies, most commonly glutamic acid decarboxylase (GAD) antibodies, it presents later in life and progresses more slowly toward insulin dependence, leading to frequent misclassification as type 2 diabetes mellitus (T2DM). LADA is not rare, accounting for 2-12% of adult-onset diabetes' however, it remains under-recognised; diagnostic delays result in prolonged hyperglycaemia, inappropriate treatment and accelerated β-cell decline. Adults with atypical glycaemic patterns, the rapid loss of glycaemic control, or coexisting autoimmune disease should be evaluated using islet autoantibodies and C-peptide testing (1). LADA exists on a spectrum from classic autoimmune diabetes to a T2DM-like phenotype, rendering diagnosis challenging, particularly in individuals retaining β-cell function at onset or presenting with obesity or insulin resistance (2). Early insulin initiation may preserve endogenous secretion (3), and guidelines support C-peptide-guided treatment decisions (4).

The present case report describes the case of a 39-year-old man with long-standing, severely uncontrolled diabetes of initially undetermined type who presented with chronic watery diarrhoea, significant unintentional weight loss, persistent hyperglycaemia and a pruritic vesicular rash, a constellation that prompted evaluation for neuroendocrine tumour, Crohn's disease and malabsorption syndromes before the underlying autoimmune diabetes was recognised.

## Case report

A 39-year-old man with diabetes of unknown type, hypertension and chronic medication non-adherence presented at the Wellstar Spalding Medical Center (Griffin, GA, USA) with a 3-month history of diffuse watery diarrhoea, nausea, vomiting and severe cramping abdominal pain. Symptoms had worsened over the preceding year, accompanied by profound unintentional weight loss from 113.4 kg (250 lb) to 57.2 kg (126 lb) over a period of 12 months, accelerating over the final 6 months. Collateral history from his mother confirmed he had been well until ~1 year earlier. He reported a pruritic vesicular rash beginning on the buttocks that spread to the left leg before partially resolving and persistent angular lip fissures. He denied fevers, chest pain, dyspnoea and substance use.

Of note, 1 year earlier, he had been hospitalised with similar symptoms, treated as colitis with intravenous fluids and a 7-day course of intravenous antibiotics and corticosteroids. His HbA1c was then >15%, and he was discharged on insulin. His family history included a father with severe uncontrolled diabetes and a brother with thyroid disease. Home medications were insulin glargine at 20 units, sliding-scale insulin, pantoprazole, ondansetron, cetirizine and hyoscyamine. He has a documented allergy to penicillins.

Upon admission he was afebrile (37.0˚C), with a blood pressure of 108/74 mmHg, a heart rate of 80 beats/min, a respiratory rate of 15/min and an oxygen saturation of 100% in room air. His height was 1.778 m and his weight was 57.2 kg, with a body mass index of 18.1 kg/m^2^, reduced from an estimated 35.9 kg/m^2^ at his pre-illness weight and representing a loss of ~50% of total body weight. He appeared thin, cachectic and ill, but non-toxic, alert and oriented. The oral mucosa was dry with bilateral angular fissures and no intra-oral lesions. A cardiac examination revealed normal heart sounds without murmurs, 2+ pulses, and no oedema; the lungs were clear. The abdomen was soft with diffuse mild-to-moderate tenderness, no rebound or guarding and normal bowel sounds. A skin examination revealed erythematous plaques over the buttocks with scattered vesicles, crusted lesions and excoriations without cellulitis; the left leg showed no active rash. Acanthosis nigricans was absent. A neurological examination was intact, and he was tearful with a depressed affect, but cooperative.

A timeline summarising the key clinical events from initial symptom onset through hospitalisation and discharge is presented in Table I.

### Investigation

The initial evaluation revealed severe hyperglycaemia (glucose, 740 mg/dl; 41.1 mmol/l); HbA1c, 14.7%; and sodium, 124 mmol/l consistent with pseudohyponatremia. β-hydroxybutyrate was mildly elevated (0.80 mmol/l) with a normal anion gap, consistent with a hyperosmolar hyperglycaemic state (HHS) without significant ketoacidosis. An endocrine workup revealed markedly reduced C-peptide (0.12 nmol/l), low glucagon (<6 ng/l) and positive islet autoantibodies (GAD-65, 33 IU/ml; islet cell, 1:8; ZnT8, 20 U/ml), confirming autoimmune aetiology. The proinsulin-to-C-peptide ratio of 6.4% indicated stressed, yet persisting β-cell function. Thyroid, parathyroid and adrenal tests yielded normal results, and 21-hydroxylase antibody was negative. Stool pancreatic elastase was low (47 µg/g). The analyses of tumour markers did not reveal any notable findings, apart from mildly elevated carcinoembryonic antigen and a borderline vasoactive intestinal peptide obtained following octreotide exposure. The levels of inflammatory markers were low, with C-reactive protein <0.3 mg/dl and faecal calprotectin at 38 µg/g. Despite positive *Saccharomyces cerevisiae* IgA and IgG, gastrointestinal evaluation revealed no definitive evidence of inflammatory bowel disease. Infectious studies (gastrointestinal multiplex polymerase chain reaction, *Clostridioides difficile* toxin, ova and parasites, human immunodeficiency virus, hepatitis serologies, QuantiFERON-TB Gold Plus) and immunoglobulin G4 were negative. The laboratory findings are summarised in Table II.

### Imaging

A computed tomography scan demonstrated diffuse sigmoid colonic wall thickening suggestive of prior colitis with non-specific small bowel wall thickening, as shown in Fig. 1. Computed tomographic enterography revealed a non-distended small bowel with mild non-specific small bowel and left colonic thickening. Magnetic resonance cholangiopancreatography revealed no biliary or pancreatic abnormality, and magnetic resonance imaging of the abdomen revealed no mass. A colonoscopic biopsy of the terminal ileum demonstrated mild non-specific active ileitis with otherwise normal colonic biopsies. Prior endoscopic biopsies revealed normal duodenal and colonic mucosa, with squamocolumnar junction inflammation and intestinal metaplasia without dysplasia.

### Differentials

Chronic watery diarrhoea, ~50% weight loss, a vesicular rash and severe hyperglycaemia prompted the evaluation of endocrine, gastrointestinal, infectious, inflammatory, and neoplastic aetiologies (Table III).

Classifying the diabetes was central. Type 2 diabetes, presumed given adult onset, obesity at diagnosis (body mass index, 35.9 kg/m^2^) and an initial non–insulin-requiring course, was excluded by C-peptide at 0.12 nmol/l, triple autoantibody positivity and progressive glycaemic failure despite escalating therapy. Classic type 1 diabetes was inconsistent: The patient remained insulin-independent for years and presented with HHS rather than ketoacidosis (β-hydroxybutyrate, 0.80 mmol/l; anion gap, 13 mmol/l; urine ketones, negative), indicating partially preserved insulin action. Adult onset, autoantibody positivity and an initial insulin-independent phase satisfy the Buzzetti consensus criteria for LADA Crohn's disease, neuroendocrine tumour, exocrine pancreatic insufficiency, celiac disease, infectious enteritis, microscopic colitis, and hyperthyroidism were each considered, with supporting and opposing evidence presented in Table III.

### Treatment

The patient was treated with intravenous isotonic fluids and an insulin infusion at 0.1 units/kg/h, with the correction of pseudohyponatremia. During an evaluation for a possible secretory neuroendocrine tumour, a short trial of octreotide transiently lowered glucose, but was discontinued when hormonal markers (glucagon, gastrin, chromogranin A) and imaging, including CT enterography and MRI of the abdomen (images not available) did not support that diagnosis.

Given biopsy-proven mild active terminal ileitis with compatible symptoms, gastroenterology concurrently treated presumptive Crohn's disease with intravenous methylprednisolone followed by an oral prednisone taper and mesalamine 2.4 g daily, with dicyclomine as needed and amitriptyline 50 mg nightly for visceral pain. Pancreatic enzyme replacement with pancrelipase was continued given the low levels of faecal elastase.

Following metabolic stabilisation, he was transitioned to subcutaneous neutral protamine Hagedorn (NPH) and regular insulin 40 units twice daily, with improved glycaemic control. Diarrhoea improved with hydration and glycaemic correction, and the rash resolved with topical emollients. He received nutritional counselling, diabetes education and home glucose monitoring instructions before discharge.

### Outcome and follow-up

At discharge after 12 days of inpatient care, glucose values were consistently 150-250 mg/dl on NPH/regular insulin, with improved appetite and energy. Outpatient follow-up was arranged for the reassessment of glycaemic control, repeat HbA1c, weight monitoring and the evaluation of gastrointestinal symptoms; however, the patient did not attend, and no long-term data were obtainable.

## Discussion

The present case report highlights the diagnostic challenge of LADA, which is frequently misclassified as T2DM. The patient described herein presented with adult-onset diabetes and a family history suggestive of T2DM and was managed accordingly until deteriorating glycaemic control prompted re-evaluation, revealing islet autoantibody positivity. Progressive β-cell failure following years of presumed T2DM is a well-described trajectory (4).

Under updated American Diabetes Association guidance, LADA is classified within the type 1 diabetes mellitus (T1DM) rubric, reflecting autoimmune β-cell destruction (5). Pathogenesis is primarily cell-mediated: Peripheral blood mononuclear cells suppress insulin secretion, and dysfunctional regulatory T-cells contribute to loss of immune tolerance (6). Patients may test positive for one or more islet autoantibodies, including GAD, IA-2A, insulin, ZnT8A, or tetraspanin 7 antibodies (7). Consensus criteria generally include adult-onset diabetes, typically after 30 years of age, islet autoantibody positivity, and an initial non-insulin-requiring course. Compared with T2DM, patients generally exhibit fewer metabolic syndrome features and more rapid C-peptide decline, although β-cell loss is typically slower than in classic T1DM (8). The patient described herein satisfied these criteria.

The antibody panel obtained, GAD-65, islet cell antibodies and ZnT8, reflects diagnostic yield and local availability. GAD antibodies are the most sensitive single marker of adult-onset autoimmune diabetes and may persist longer than several other islet autoantibodies. Islet cell antibodies, although less standardized, provide complementary evidence of islet-directed autoimmunity. ZnT8 antibodies may provide incremental diagnostic sensitivity and contribute to identification of multiple-autoantibody positivity, a phenotype associated with more rapid β-cell decline and earlier insulin requirement. IA-2A was not obtained; its inclusion could have further refined immunologic characterization as IA-2A positivity has been shown to be associated with a more rapidly progressive phenotype and a higher prevalence of coexisting celiac disease (9,10). Nonetheless, positivity for three distinct islet autoantibodies together with a C-peptide of 0.12 nmol/l strongly supports an autoimmune basis, as multiple-autoantibody positivity provides greater specificity than any single marker alone.

As gastrointestinal and cutaneous manifestations predominated rather than classical hyperglycaemic symptoms, several alternative diagnoses were entertained. Diagnostic confusion arose as diabetes had already been established and presumed to be type 2; consequently, the constellation of cachexia, diarrhoea and rash was initially interpreted as a separate coexisting process, rather than potentially related to profound insulin deficiency (11). An abrupt classic T1DM presentation was less consistent with the multi-year non-insulin-requiring course and presentation with HHS rather than ketoacidosis. The level of β-hydroxybutyrate was only 0.80 mmol/l and the anion gap was normal, suggesting sufficient residual insulin action to suppress substantial ketogenesis. As LADA lies along the autoimmune T1DM spectrum, the distinction largely reflects the tempo of β-cell loss and the timing of insulin dependence rather than fundamentally different pathology.

A neuroendocrine tumour was considered due to weight loss, secretory-type diarrhoea, mildly elevated carcinoembryonic antigen and a transient response to octreotide. However, glucagon, gastrin and chromogranin A levels were normal, imaging demonstrated no corresponding lesion, and the octreotide response could reflect its anti-secretory effects rather than tumour-directed activity. Hyperglycaemia was also better accounted for by insulin deficiency than by counter-regulatory hormone excess. Crohn's disease was considered due to positive anti-*Saccharomyces* antibodies, crampy abdominal pain, prior colitis, and biopsy-proven mild ileitis and was treated empirically. However, these antibodies have limited positive predictive value in isolation, the levels of inflammatory markers were low, and no skip lesions, ulceration, granulomata, or transmural inflammation were identified. Malabsorption syndromes, including celiac disease, were also considered due to weight loss, diarrhoea, low levels of faecal elastase and angular cheilitis; however, normal total IgA, negative gliadin antibodies, normal duodenal histology and the absence of steatorrhea argued against celiac disease.

A critical caveat applies when interpreting the clinical improvement. As empirical corticosteroid and mesalamine therapy was administered concurrently with insulin, improvement cannot be attributed to glycaemic correction alone. Several findings nevertheless favour a predominantly metabolic explanation: Faecal calprotectin and C-reactive protein levels were low, colonoscopic and histologic abnormalities were mild and non-specific without diagnostic features of Crohn's disease, and the degree of cachexia was consistent with profound insulin deficiency.

Gastrointestinal manifestations are not hallmark features of LADA, although autoimmune comorbidities, including thyroid disease, celiac disease and dermatitis herpetiformis, have been reported, reflecting shared autoimmune predisposition rather than a direct causal relationship (9,11). Reported associations are summarized in Table IV. Despite chronic diarrhoea and a vesicular rash, evaluation did not confirm celiac disease or another specific enteropathy in the patient in the present case report.

Progression to HHS is atypical; however, it has been reported and may occur when progressive β-cell destruction produces severe insulin deficiency without sufficient absolute insulin deficiency to generate marked ketogenesis (12).

The mechanism of chronic diarrhoea also merits consideration. Sustained hyperglycaemia may produce gastrointestinal dysfunction through altered autonomic regulation and intestinal motility. The low faecal elastase of 47 µg/g falls within a range conventionally interpreted as severe exocrine pancreatic insufficiency; however, several considerations argue against primary pancreatic disease in this case. Faecal elastase-1 measures concentration, rather than total output and can yield falsely low values in watery stool due to dilution. In one series, 11% of diarrheal samples with values <200 µg/g normalized following correction for water content, supporting repeat testing on a formed stool specimen before definitive interpretation (13,14). Imaging, including MRCP (images not available) demonstrated no ductal dilatation, calcification, pancreatic atrophy, or mass and steatorrhea was absent. Reduced exocrine pancreatic function has also been described in longstanding insulin-deficient diabetes (15), potentially reflecting the loss of insulin-mediated trophic effects on acinar tissue. Thus, the low elastase in the present case may reflect stool dilution and diabetes-associated exocrine hypofunction, rather than independent pancreatic pathology.

At a pathophysiologic level, the autoimmune nature of LADA is consistent with predominantly cell-mediated β-cell injury, including impaired immune tolerance and abnormalities in regulatory T-cell function (15). This progressive autoimmune destruction explains the transition from an initially non–insulin-requiring phenotype to profound endogenous insulin deficiency.

C-peptide declines more slowly in LADA than in classic T1DM, although generally more rapidly than in T2DM. Management can therefore be guided in part by residual β-cell function: C-peptide values <0.3 nmol/l support treatment as insulin-deficient T1DM, values of 0.3-0.7 nmol/l represent an intermediate range in which insulin should be considered alongside other therapies, and values >0.7 nmol/l may permit a modified T2DM-based strategy with continued monitoring (8,15,16). At 0.12 nmol/l, the patient described herein had profound endogenous insulin deficiency and was clearly insulin-requiring. Agents, including dipeptidyl peptidase-4 (DPP-4) inhibitors, glucagon-like peptide-1 (GLP-1) receptor agonists and thiazolidinediones have been investigated for potential β-cell-preserving effects, whereas sulfonylureas may accelerate the deterioration of β-cell function and sodium-glucose transport protein 2 (SGLT2) inhibitors require caution due to the risk of ketoacidosis (17).

Compared with T2DM, LADA has been associated with poorer glycaemic control and differences in long-term microvascular outcomes, while higher GAD antibody titres have been associated with cardiovascular events in some cohorts (18). These observations support maintaining a high index of suspicion for autoimmune diabetes in adults whose clinical course is atypical for T2DM and considering islet autoantibody assessment when classification remains uncertain (19-21).

The present case report has certain limitations which should be mentioned. The absence of longitudinal follow-up precludes the documentation of progressive β-cell failure and sustained symptom resolution. As immunosuppression was administered concurrently with insulin, their relative contributions cannot be separated. As the rash was not biopsied, dermatitis herpetiformis cannot be formally excluded, and IA-2A and stimulated C-peptide were not obtained.

In conclusion, the present case report emphasizes that profound insulin deficiency may present with systemic manifestations that mimic malignancy, inflammatory bowel disease, or malabsorptive disorders and may therefore be overlooked when diabetes has already been presumed to be type 2.

## Figures and Tables

**Figure 1:**
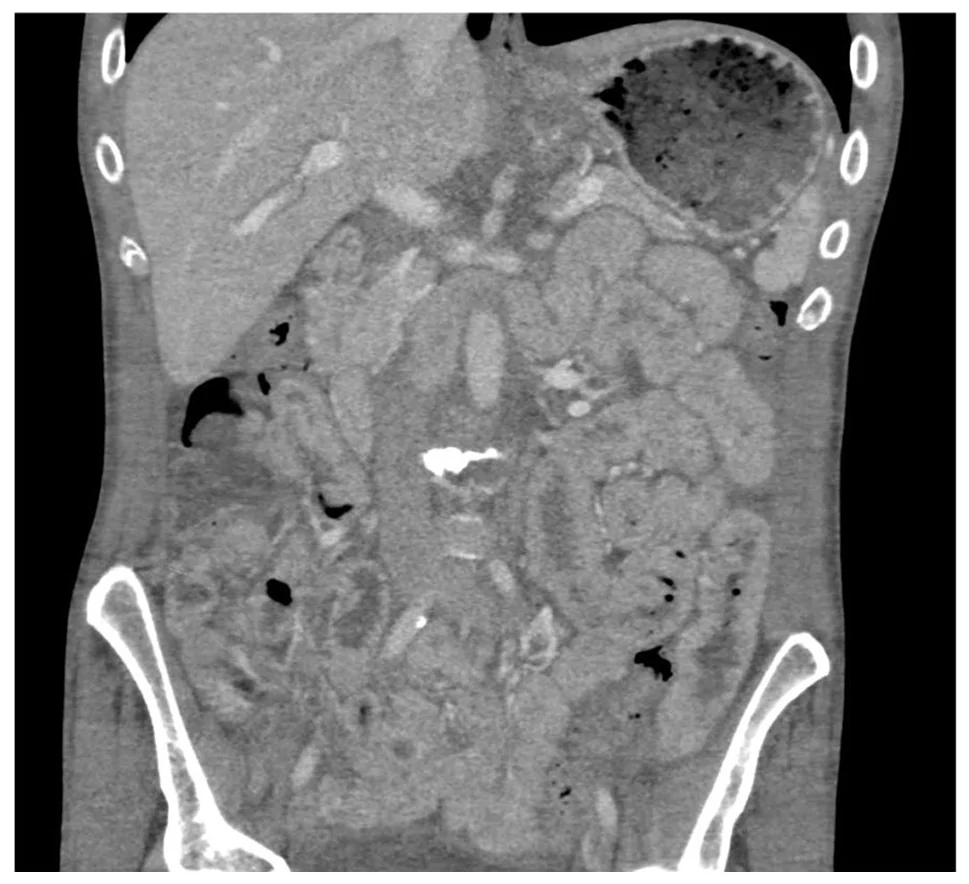
Computed tomography scan of the abdomen/pelvis with contrast illustrating diffuse intramural thickening of the colon identified in the sigmoid region; this may represent sequelae of colitis. Additionally, diffuse intramural thickening of the small bowel loops was noted.

**Table I:** Timeline of key clinical events.

Timeline	Clinical event
Age, 30s	Onset of diabetes symptoms; classified as type 2 diabetes mellitus; started on oral hypoglycaemic agents
1 year before index admission	First hospitalisation: one month of crampy abdominal pain, nausea, vomiting, diarrhoea; treated for colitis with IV fluids, antiemetics, analgesics, 7-day course of IV antibiotics and corticosteroids; HbA1c >15%; discharged on insulin regimen (glargine 20 units plus sliding scale)
6 months befor index admission	Acceleration of progressive unintentional weight loss (113.4 kg → 57.2 kg; 250 lb → 126 lb over 12 months, with marked acceleration over the final 6 months)
3 months before index admission	Onset of diffuse watery diarrhoea, worsening nausea, vomiting, and severe cramping abdominal pain; pruritic vesicular rash appears on buttocks, spreads to left leg, then partially resolves; persistent angular fissures at lip corners
Day 0 (Index admission)	Admitted with hyperosmolar hyperglycaemic state: glucose 740 mg/dl (41.1 mmol/l), HbA1c 14.7%, β-hydroxybutyrate 0.80 mmol/l, normal anion gap, sodium 124 mmol/l (pseudohyponatremia); started on IV insulin infusion at 0.1 units/kg/h and isotonic fluids
Days 1-3	Endocrine workup: C-peptide 0.12 nmol/l, glucagon <6 ng/l, proinsulin-to-C-peptide ratio 6.4%; islet autoantibodies positive (GAD-65 33 IU/ml, ICA 1:8, ZnT8 20 U/ml); thyroid, parathyroid, and adrenal studies normal; faecal elastase 47 µg/g; tumour markers unremarkable except mildly elevated CEA; short trial of octreotide initiated (transiently lowered glucose; discontinued when hormonal workup did not support neuroendocrine tumour)
Days 3-5	CT abdomen/pelvis: diffuse sigmoid wall thickening suggestive of prior colitis, nonspecific small bowel wall thickening; CT enterography: nondistended small bowel with mild non-specific thickening; MRCP and MRI abdomen: no biliary, pancreatic, or mass lesion
Days 5-7	Colonoscopy with biopsy: Mild nonspecific active terminal ileitis, otherwise normal colonic biopsies; stool studies (GI multiplex PCR, C. difficile toxin, ova and parasites): negative; Saccharomyces IgA/IgG: positive; faecal calprotectin 38 µg/g; CRP <0.3 mg/dl
Days 5-10	Gastroenterology commenced empirical therapy for presumptive Crohn's disease: IV methylprednisolone followed by oral prednisone taper, mesalamine 2.4 g daily, dicyclomine as needed, amitriptyline 50 mg nightly; pancrelipase continued
Days 7-10	Transitioned from IV insulin to subcutaneous NPH and regular insulin 40 units twice daily; diarrhoea improved with hydration and glycaemic control; rash resolved with topical emollients; nutritional counselling, diabetes education, and home glucose monitoring instructions provided
Discharge	Diagnosis confirmed: LADA presenting with hyperosmolar hyperglycaemic state; glucose values 150-250 mg/dl on NPH/regular insulin; improved appetite and energy; discharged on NPH/regular insulin 40 units twice daily and supportive medications
Post-discharge	Outpatient follow-up arranged for reassessment of glycaemic control, repeat HbA1c, weight monitoring, and evaluation of GI symptoms; patient did not attend; no long-term data obtainable

CEA, carcinoembryonic antigen; CRP, C-reactive protein; CT, computed tomography; GAD-65, glutamic acid decarboxylase 65; GI, gastrointestinal; HbA1c, glycated haemoglobin; ICA, islet cell antibodies; IV, intravenous; LADA, latent autoimmune diabetes in adults; MRCP, magnetic resonance cholangiopancreatography; MRI, magnetic resonance imaging; NPH, neutral protamine Hagedorn; PCR, polymerase chain reaction; ZnT8, zinc transporter 8.

**Table II:** Summary of laboratory findings and infectious aetiology workup.

Test	Result (SI units)	Reference range (SI units)	Interpretation
Sodium	124 mmol/l	135-145 mmol/l	Low
Potassium	3.8 mmol/l	3.5-5.0 mmol/l	Normal
Chloride	96 mmol/l	98-107 mmol/l	Low
Bicarbonate	19 mmol/l	23-30 mmol/l	Low
WBC	5.92x10^9^/l	4-10x10^9^/l	Normal
Glucose	41.1 mmol/l	<11.1 mmol/;	High
Anion Gap	13 mmol/l	12-16 mmol/;	Normal
Beta-hydroxybutyrate	0.80 mmol/l	<0.5 mmol/l	High
HbA1c	14.7%	<5.7%	High
C-reactive protein	<0.3 mg/dl	<0.5 mg/dl	Normal
Albumin	3.3 g/dl	3.5-5.0 g/dl	Low
Fecal calprotectin	38 µg/g	<50 µg/g	Normal
Glucagon	<6 ng/l	11-78 ng/l	Low
C-peptide	0.12 nmol/l	0.26-1.28 nmol/l	Low
Proinsulin	7.7 pmol/l	≤18.8 pmol/l	Normal
Proinsulin-to-C-peptide ratio	6.4%	-	Residual β-cell function
GAD-65 Ab	33 IU/ml	<5 IU/ml	High
Islet Cell Ab	1:8	<1:4	High
ZnT8 Ab	20 U/ml	<10 U/ml	High
Calcitonin	<2 ng/l	≤10 ng/l	Normal
Chromogranin A	Normal	Per laboratory reference	Normal
Gastrin	Normal	Per laboratory reference	Normal
CA 19-9	30.5 U/ml	<47 U/ml	Normal
CEA	7.9 ng/ml	<3 ng/ml	High
IgA (total)	3.99 g/l	0.7-4.0 g/l	Normal
AFP	1.9 ng/ml	<10 ng/ml	Normal
VIP	47 ng/l	<36 ng/l	High
Pancreatic elastase (stool)	47 µg/g	>200 µg/g	Low
NSE	8 ng/ml	<14 ng/ml	Normal
IGF-1	52 µg/l	53-331 µg/l	Low
ACTH	18 ng/l	6-50 ng/l	Normal
Prolactin	Normal	Per laboratory reference	Normal
Gliadin IgA	2 U/ml	<7 U/ml	Normal
ANA	Negative	Negative	Normal
ANCA	Negative	Negative	Normal
21-Hydroxylase Ab	Negative	Negative	Normal
Immunoglobulin G4	Negative	Per laboratory reference	Normal
TSH	2.75 mIU/l	0.4-4.0 mIU/l	Normal
PTH	25 pg/ml	10-55 pg/ml	Normal
Vitamin D 25-OH	67.5 nmol/l	75-250 nmol/l	Low
Vitamin B12	729 ng/l	139-619 ng/l	High
Folate	>45 nmol/l	7-34 nmol/l	High
Zinc	5.96 µmol/l	9.2-20 µmol/l	Low
Saccharomyces cerevisiae Ab IgA	66.8 U/ml	>25 U/ml	Elevated
Saccharomyces cerevisiae Ab IgG	67.5 U/ml	>30 U/ml	Elevated
HIV 1/2 Antigen/Antibody	Non-reactive	Non-reactive	Normal
Hepatitis A (IgM) Ab	Non-reactive	Non-reactive	Normal
Hepatitis B Core (IgM) Ab	Non-reactive	Non-reactive	Normal
Hepatitis B Surface Ag	Non-reactive	Non-reactive	Normal
Hepatitis C Ab	Non-reactive	Non-reactive	Normal
GI Multiplex PCR	No pathogens detected	Negative	Normal
Clostridioides difficile toxin PCR	Negative	Negative	Normal
Stool ova and parasites	Negative	Negative	Normal
Stool leukocytes	Negative	Negative	Normal
QuantiFERON-TB Gold Plus	Negative	Negative	Normal

Ab, antibody; ACTH, adrenocorticotropic hormone; AFP, alpha-fetoprotein; ANA, antinuclear antibody; ANCA, antineutrophil cytoplasmic antibody; CEA, carcinoembryonic antigen; GAD-65, glutamic acid decarboxylase 65; GI, gastrointestinal; HbA1c, glycated haemoglobin; HIV, human immunodeficiency virus; IgA, immunoglobulin A; IgG, immunoglobulin G; IGF-1, insulin-like growth factor 1; NPH, neutral protamine Hagedorn; NSE, neuron-specific enolase; PCR, polymerase chain reaction; PTH, parathyroid hormone; TB, tuberculosis; TSH, thyroid-stimulating hormone; VIP, vasoactive intestinal peptide; WBC, white blood cell count; ZnT8, zinc transporter 8.

**Table III:** Structured differential diagnosis: Supporting and opposing evidence.

Diagnosis	Supporting evidence	Opposing evidence (Refs.)	Conclusion (Refs.)
Type 2 diabetes mellitus	Adult onset; obesity at diagnosis (BMI 35.9 kg/m^2^); paternal history of severe diabetes; initial non-insulin-requiring course	C-peptide 0.12 nmol/l (reference, 0.26-1.28); triple islet autoantibody positivity (GAD-65 33 IU/ml, ICA 1:8, ZnT8 20 U/ml); progressive glycaemic failure despite escalating therapy; no metabolic syndrome features at presentation	Excluded
Classic type 1 diabetes mellitus	Triple islet autoantibody positivity; near-absent C-peptide; insulin dependence; sibling with thyroid disease	Obesity at diagnosis; documented non-insulin-requiring interval of several years; hyperosmolar hyper-glycaemic state rather than ketoacidosis (β-hydroxybutyrate 0.80 mmol/l, anion gap 13 mmol/l, urine ketones negative, urine glucose 3+), indicating partially preserved insulin action; proinsulin-to-C-peptide ratio 6.4%	Excluded; initial insulin-independent phase discriminates LADA per Buzzetti consensus criteria (7)
Crohn's disease	ASCA IgA 66.8 U/ml and IgG 67.5 U/ml (both elevated); crampy abdominal pain with weight loss; CT small-bowel and sigmoid wall thickening; mild nonspecific active ileitis on terminal ileal biopsy; prior admission treated as colitis; empirical corticosteroid and mesalamine therapy administered	Faecal calprotectin 38 µg/g and CRP <0.3 mg/dl, both normal; no ulceration, cobblestoning, or skip lesions at colonoscopy; no granulomas or chronic architectural distortion; random colonic biopsies normal on two occasions; ANCA negative; CT enterography revealed non-specific thickening without stricture or fistula; ASCA has limited positive predictive value in isolation	Not established; empirical treatment given on the basis of biopsy-proven mild ileitis. Because immunosuppression was administered concurrently with insulin, relative contribution of each to symptom improvement cannot be separated
Neuroendocrine tumour (VIPoma, carcinoid, gastrinoma, glucagonoma)	Secretory-pattern high-volume watery diarrhoea; ~50% weight loss; VIP mildly elevated at 47 ng/l (reference, <36); CEA 7.9 ng/ml (reference, <3); transient symptomatic response to octreotide	Glucagon <6 ng/l, gastrin normal, chromogranin A normal, calcitonin <2 ng/l, NSE 8 ng/ml, CA 19-9 30.5 U/ml, AFP 1.9 ng/ml, ACTH 18 ng/l, proinsulin 7.7 pmol/l all normal or non-diagnostic; no flushing, peptic ulceration, or necrolytic migratory erythema; CT, CT enterography, MRCP, and MRI abdomen without pancreatic or luminal lesion	Excluded; modest VIP elevation attributed to dehydration, catabolic state, and prior octreotide exposure (somatostatin receptor imaging not obtained; repeat VIP after adequate washout not performed)
Exocrine pancreatic insufficiency/ malabsorption	Chronic diarrhoea; ~50% weight loss; faecal elastase 47 µg/g (severe range, reference >200); angular cheilitis; zinc 5.96 µmol/l (low); vitamin D 25-OH 67.5 nmol/l (low); albumin 3.3 g/dl; IGF-1 52 µg/l (low)	No steatorrhea; MRCP revealed no ductal stone or biliary abnormality; CT without pancreatic parenchymal change; no history of pancreatitis, alcohol, or tobacco use; IgG4 negative; vitamin B12 729 ng/l and folate >45 nmol/l both elevated, arguing against generalised small-bowel malabsorption; watery stool falsely lowers faecal elastase through dilution (13,14)	Not primary pancreatic disease; low elastase attributed to stool dilution and loss of islet–acinar trophic support from profound insulinopenia; pancrelipase continued empirically
Celiac disease/ dermatitis herpetiformis	Chronic diarrhoea; weight loss; angular cheilitis; pruritic vesicular buttock rash	Normal duodenal histology at two separate endoscopies; anti-gliadin IgA 2 U/ml (negative); total IgA 3.99 g/l (normal), excluding IgA-deficiency false negatives	Excluded (tissue transglutaminase IgA not obtained; rash not biopsied, so dermatitis herpetiformis cannot be formally excluded)
Infectious enteritis	Chronic diarrhoea; prior ciprofloxacin and metronidazole exposure; pro-found weight loss	GI multiplex PCR negative for all pathogens; stool ova and parasites negative; Clostridioides difficile toxin PCR negative; stool leukocytes negative; HIV-1/2 antigen–antibody non-reactive; hepatitis A IgM, hepatitis B core IgM, HBsAg, and HCV non-reactive; QuantiFERON-TB Gold Plus negative	Excluded
Microscopic colitis	Chronic watery diarrhoea	Random colonic biopsies normal on two separate occasions, with no lymphocytic or collagenous changes	Excluded
Hyperthyroidism	Diarrhoea with weight loss	TSH 2.75 mIU/l (normal)	Excluded

ACTH, adrenocorticotropic hormone; AFP, alpha-fetoprotein; ANCA, antineutrophil cytoplasmic antibody; ASCA, anti-Saccharomyces cerevisiae antibody; BMI, body mass index; CEA, carcinoembryonic antigen; CRP, C-reactive protein; CT, computed tomography; GAD-65, glutamic acid decarboxylase 65; GI, gastrointestinal; HBsAg, hepatitis B surface antigen; HCV, hepatitis C virus; HIV, human immunodeficiency virus; ICA, islet cell antibodies; IgG4, immunoglobulin G4; IGF-1, insulin-like growth factor 1; LADA, latent autoimmune diabetes in adults; MRCP, magnetic resonance cholangiopancreatography; MRI, magnetic resonance imaging; NSE, neuron-specific enolase; PCR, polymerase chain reaction; T1DM, type 1 diabetes mellitus; TSH, thyroid-stimulating hormone; VIP, vasoactive intestinal peptide; ZnT8, zinc transporter 8.

**Table IV:** Reported associations between autoimmune disorders and LADA.

Authors	Linked autoimmune disorder	Evidence of association with LADA	Overlapping pathophysiology	Autoantibodies/immune markers reported	(Refs.)
Santoso, *et al*	Thyroid autoimmune disease (Hashimoto thyroiditis, Graves disease)	Thyroid dysfunction confers approximately 1.88-fold higher odds of LADA compared with T2DM	Shared genetic susceptibility within the HLA/MHC region with over-lapping B-cell and T-cell mediated immune mechanisms	Frequency of thyroid peroxidase or thyroglobulin antibody positivity significantly higher in LADA cohorts than in T2DM or controls	(10)
Kucera, *et al*	Gastrointestinal auto-immunity, including celiac and gluten-related disease	Increased frequency of gliadin and endomysial antibodies in LADA compared with T2DM	Shared immuno-genetic background affecting both gut and islet autoimmunity	Anti-gliadin IgA and IgG, and occasionally endomysial antibodies, in addition to islet autoantibodies	(11)
Gougourelas, *et al*	Other organ-specific autoimmune disease, including inflammatory bowel disease	Inflammatory bowel disease associated with increased odds of LADA (odds ratio approximately 1.78)	LADA may represent one manifestation of a broader tendency toward autoimmunity; shared HLA/MHC and other immuno-genetic loci with environmental triggers may underlie this polyautoimmunity	Islet autoantibodies (GAD, IA-2, sometimes ZnT8) withadditional nonpancreatic autoantibodies such as thyroid or celiac markers	(8)

GAD, glutamic acid decarboxylase; HLA, human leukocyte antigen; IA-2, insulinoma-associated antigen 2; LADA, latent autoimmune diabetes in adults; MHC, major histocompatibility complex; T2DM, type 2 diabetes mellitus; ZnT8, zinc transporter 8.

## Data Availability

The data generated in the present study may be requested from the corresponding author.
